# Prospective Five-Year Follow-Up of Patients with Schizophrenia Suspected with Parkinson's Disease

**DOI:** 10.1155/2022/2727515

**Published:** 2022-05-26

**Authors:** Asako Yoritaka, Tetsuo Hayashi, Keiko Fusegi, Rie Inami, Nobutaka Hattori

**Affiliations:** ^1^Department of Neurology, Juntendo University Koshigaya Hospital, Saitama, Japan; ^2^Department of Neurology, Juntendo University School of Medicine, Tokyo, Japan; ^3^Department of Psychiatry, Juntendo University Koshigaya Hospital, Saitama, Japan

## Abstract

**Objective:**

It is difficult to distinguish patients with schizophrenia with neuroleptic-induced parkinsonism (NIP) from those with existing idiopathic Parkinson's disease when their striatal dopamine transporter uptake is reduced. There is a possibility of misdiagnosis of Parkinson's disease in patients with schizophrenia as schizophrenia with NIP, which leads to inappropriate treatment. This prospective study aimed at determining the underlying pathophysiology using detailed clinical and psychological assessments.

**Methods:**

We enrolled six patients with schizophrenia who had parkinsonism and were diagnosed with Parkinson's disease according to the Movement Disorder Society Clinical Diagnostic Criteria, except for the fifth absolute exclusion criteria.

**Results:**

Five patients had been treated with neuroleptics for 20 years. One patient refused treatment for schizophrenia. All patients had impaired cognitive function at enrolment, olfactory dysfunction, and constipation. All patients were treated with dopaminergic therapy, and their parkinsonism substantially improved; one woman in her 40s experienced a wearing-off effect and dyskinesia. The uptake of dopamine transporter in the striatum decreased by 13%/year during the study period.

**Conclusion:**

Some patients with schizophrenia and parkinsonism benefit from dopaminergic therapy. Some of these patients may also exhibit Lewy pathology.

## 1. Introduction

Neuroleptic-induced parkinsonism (NIP) is a complication of dopamine receptor block or a decrease in dopamine that induces parkinsonism. Differentiation between NIP and idiopathic Parkinson's disease (PD) is difficult in terms of the reduction in striatal dopamine transporter uptake in patients with schizophrenia. Imaging with specific striatal dopamine transporter (DAT) ligands for single-photon emission computed tomography (SPECT) provides a marker for presynaptic nigral neurodegeneration. Striatal presynaptic dopaminergic hyperactivity is among the most widely replicated results in neurochemical imaging studies of schizophrenia [1]. The cause of schizophrenia remains unclear, although evidence suggests that it may be produced by an excess transmission of dopamine in selected brain regions [2]. Hence, reduced striatal DAT is typical for PD and recommended for the differentiation between idiopathic and drug-induced parkinsonism.

However, Galoppin et al. [3] reported that striatal [11C] (+) dihydrotetrabenazine results in NIP extend previous DAT imaging studies using SPECT [4] and that striatal binding in NIP is divided into spared and low vesicular monoamine transporter type 2 (VMAT2), where PD-like bindings would be clinically indistinguishable from PD. We aimed at determining whether parkinsonism in such patients is accompanied by PD or whether it is due to the degeneration of striatal neurons associated with schizophrenic pathology. We examined patients with a long history of schizophrenia who developed parkinsonian symptoms in their later life with reduced striatal DAT uptake. We followed their parkinsonian symptoms, psychogenic symptoms, cognitive functions, and DAT scan results.

## 2. Methods

### 2.1. Patients

We initially assessed 20 patients who received treatment for schizophrenia and manifested parkinsonism between April 2016 and March 2021. They were examined by a certified neurologist at the neurology clinic at Juntendo University Koshigaya Hospital and had their DAT scan taken. The inclusion criteria were as follows: diagnosis of schizophrenia according to the Diagnostic and Statistical Manual of Mental Disorders IV; parkinsonism that conformed to the criteria of the Movement Disorder Society (MDS), except for the fifth absolute exclusion criterion (treatment with a dopamine receptor blocker or a dopamine-depleting agent in a dose and time-course consistent with drug-induced parkinsonism) [5]; and reduced striatal uptake on DAT scans. The exclusion criterion was high disease activity of schizophrenia. Eight patients met the criteria of the MDS, except for the fifth absolute exclusion criterion, but two patients refused to participate in the study.

This prospective study was approved by the Juntendo Koshigaya Hospital Institutional Ethics Committee (Koshigaya 28-1), and we obtained written informed consent for publication from all patients.

We performed the following assessments within 3 months of enrolment of every patient and annually thereafter: the Mini Mental Scale Examination (MMSE-J), Montreal Cognitive Assessment-Japan (MOCA-J), Neuropsychiatric Inventory (NPI), Frontal Assessment Battery (FAB), Wechsler Adult Intelligence Scale-III (WAIS), noise pareidolia test (pareidolic illusions using meaningless visual noise stimuli [6]), MDS-Unified Parkinson's Disease Rating Scale (UPDRS), Parkinson's disease Questionnaire-39-J, and the Japanese version of the REM (rapid eye movement) sleep behaviour disorder questionnaire (RBDQ-JP) [7]. An olfactory test (OSIT-J test) [8] was conducted at enrolment.

### 2.2. SPECT Imaging

[^123^I]FP-CIT (167MBq) was injected intravenously in a volume of 2 ml. Scans were obtained at 3 h after tracer injection, and the total scan time was 30 min. The specific binding ratio (SBR) was calculated using DATview software (AZE, Tokyo). A DAT scan was obtained every 2 years.

## 3. Results

The clinical characteristics of the six enrolled patients (age 66.7 ± 14.5: mean ± standard deviation; two male, four female) are shown in Table 1. All patients were followed up until March 2021. We could not conduct any statistical analysis due to the small sample size.

**Figure 1:**
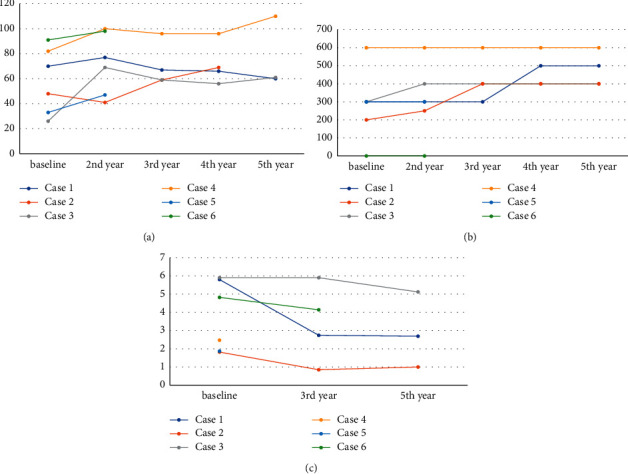
Follow-up findings. (a) Total Movement Disorder Society-Unified Parkinson's Disease Rating Scale (MDS-UPDRS), (b) daily levodopa dose, and (c) average specific binding ratio of dopamine transporter imaging.

There were no genetic abnormalities (PARK 1, 2, 6, 7, 8) in cases 1, 2, 3, and 4, and case 5 or 6 did not agree with genetic testing. Four out of the six enrolled patients were followed up for more than 5 years. The interval between the diagnosis of schizophrenia and the diagnosis of parkinsonism was 12–43 years. Case 2 reported that she had been “sensitive to spiritual things” since her 20s and that she had assumed this was a “special power” and had therefore not consulted any doctors until she was 56 years old. Three patients (1, 3, 6) were treated with D2 receptor antagonists, while the other patients met all the MDS criteria. All patients received parkinsonian drugs (levodopa, dopamine agonist, entacapone, zonisamide), which had prominent effects on their parkinsonism. Psychological deterioration or exacerbation was not observed with anti-parkinsonian treatment in those whose daily doses were increased due to the worsening of parkinsonism (Figure 1(b)). We did not prescribe any additional anticholinergic drugs for parkinsonism at our clinic. In Cases 1 and 2, the manifested NPI subitems were the same at baseline and at the end of the study (Table 2). Case 3 expressed anxiety at the end of the study; however, it was confirmed by their caregiver that the patient was newly experiencing knee complications and had just become aware of lumps in her chest, and was therefore worried that she might have cancer. This finding was therefore not related to the patient's anti-parkinsonian medication. None of the patients had changed their schizophrenic treatments. Cognitive function was low at enrolment with the exception of Case 1. The patients' executive function was also severely low. The followed-up noise pareidolia test results revealed no progression to visual hallucination or deterioration of visual cognition except in Case 2. Case 2 showed significant deterioration of cognition, visual illusions of noise on the pareidolia test, and significant worsening of the MDS-UPDRS score over the course of the study period (Figure 1(a)).

Parkinsonian treatment improved the modified Hoehn and Yahr stage from diagnosis to after the first treatment in Case 1 (stage 5 to 3), Case 2 (stage 4 to 3), and Case 3 (stage 3 to 2). The youngest patient (Case 1) experienced a wearing-off and dyskinesia after 2 years of parkinsonian treatment. Parkinsonism in our patients was relatively symmetrical. Olfactory function was severely disturbed in all examined patients, and Cases 4 and 6 exhibited RBD signs, determined using the questionnaire.

**Figure 2:**
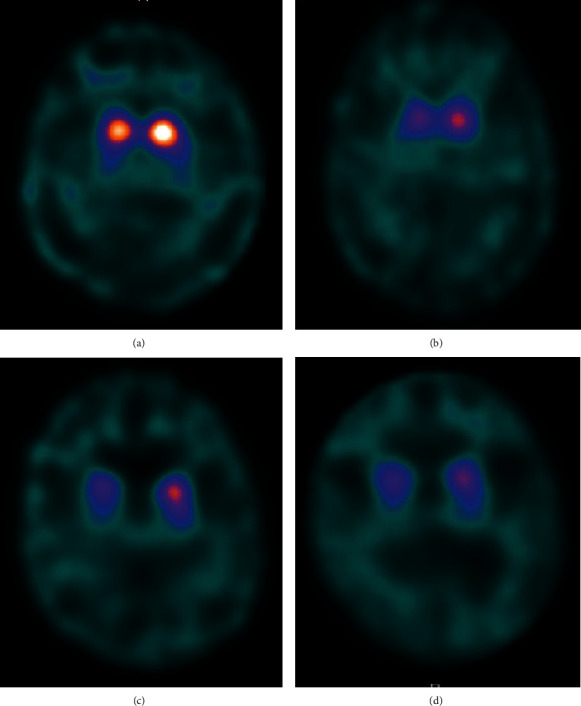
Dopamine transporter imaging. (a) Baseline imaging of Case 1, (b) 5th year imaging of Case 1, (c) baseline imaging of Case 2, and (d) 5th-year imaging of Case 2.

The average and 95% confidence interval of SBR recorded in the Japanese healthy control database [9] was as follows: women in their 40s, 9.35 (6.71–10.62); men in their 50s, 8.05 (5.41–11.61); women in their 70s, 7.06 (4.42–10.92); and men in their 80s, 6.61 (3.95–13.25). The SBR in the striatum on the DAT scans decreased gradually (Figure 1(c), Figure 2), and the percentage changes in the SBR ratio from baseline to follow-up were as follows: Case 1, right (rt) 54.8%; left (lt) 52.5%/5 years; Case 2, rt 28.0%, lt 61.7%/5 years; and Case 3, rt 15.5%, lt 11.1%/4 years. The annual decline rate was calculated as 13% per year in the first 2 years and 5% to 6% per year in the subsequent 3 years. There were no findings of cerebrovascular changes in our patients.

## 4. Discussion

Patients with schizophrenia who manifest parkinsonism are usually assumed to be drug-induced instead of an actual Parkinson's disease. Since the launch of second-generation antipsychotic drugs, the incidence of drug-induced parkinsonism has been reduced [10]. However, some studies report otherwise [11, 12].

In the current study, decreasing DAT availability was associated with progressive parkinsonism in some schizophrenia patients. The gradual worsening of parkinsonism in these patients was in line with the MDS clinical criteria, and they were responsive to dopamine therapy. The dopamine therapy in this study was not accompanied by psychological exacerbation, and the dose of dopamine was increased during the study period. Symptomatically, asymmetry was not observed, as in an earlier report that NIP by D2 receptor antagonist is typically bilateral [10]. However, the SBR on the DAT scan was found to be asymmetric in this study.

The annual decline rate of DAT binding in the striatum has been reported to be 5%–11% in patients with PD and 0.8% in control subjects [13–15]. In our study, the annual decline rate was calculated as 5% to 13% per year. The decrease rate in SBR of elderly controls has been found to be smaller than that of younger controls [16,17]. Our youngest patient, Case 1, exhibited a prominent decrease in SBR compared to the controls in earlier reports, and even in our elderly cases, the decline was higher than that in those controls [13–15]. Also, the density of putaminal tyrosine hydroxylase and immunohistochemistry in PD patients showed a modest loss during year 1–3 after diagnosis, a rapid decline through years 4–5, and relatively stable levels thereafter [18]. When parkinsonism in some cases worsened with decreasing SBR, the dose of dopaminergic therapy was increased.

Our patients who underwent genetic testing did not exhibit any genetic abnormalities. However, two of three patients carrying both a parkin and a PINK1 mutations have been reported to have schizophrenia, and patients with both mutations are younger at onset of Parkinsonism than those with the same parkin mutation alone [19]. In a young patient with *α*-synuclein multiplication, the major onset symptom was schizophrenia [20], and another young patient with a missense variation of *α*-synuclein exhibited schizophrenic symptoms that severely impaired his cognitive function [21]. A recent cohort study from Finland reported that the odds ratio for PD based on the International Classification of Diseases-10 after a diagnosis of schizophrenia was 4.68 (1.35–16.31, *P* = 0.02) [22]. The increased PD risk in patients with schizophrenic spectrum disorders could be related to neuroleptic exposure to a chronic hypodopaminergic state after tonic-phasic dopaminergic fluctuations in active schizophrenic spectrum disorder [22]. Moreover, genome-wide association studies have indicated common molecular genetic mechanisms between schizophrenia and PD [23].

We estimated that some of our patients would also have PD. The patients who had the onset of their parkinsonism at an advanced age showed deterioration of motor symptoms. However, one patient (Case 1) whose parkinsonism started at a young age received dopaminergic therapy and showed significant improvement in their symptoms, including cognition.

Our longitudinal cohort study of idiopathic PD revealed that 42.5% of our patients experienced psychosis, mainly visual hallucinations, as well as a false sense of presence, illusions, and auditory hallucinations. An earlier Kaplan–Meier curve analysis has revealed that psychosis occurs at a 2.0% rate during the 2nd year, a 3.7% rate during the 4th year, a 7.4% rate during the 6th year, and a 12.6% rate during the 8th year [24]. Hazard ratios of psychosis were not significant in the cumulative dose of total dopamine agonist and cumulative dose of trihexyphenidyl, amantadine, or selegiline until the onset of psychosis, although many patients in that study had to discontinue or decrease the doses of drugs other than levodopa [24]. According to a postmarketing survey, the side effects of psychosis are observed in 10.1% of patients prescribed ropinirole CR (controlled release) [25], 3.9% of patients taking pramipexole-ER (extended release) [26], and 8.5% of patients using rotigotine transdermal patches [27]. In the current study, the NPI did not reveal hallucinations in Case 2 after the treatment of parkinsonism, although the noise pareidolia test had brought our attention to the patient's visual misperceptions. The severe deterioration of cognitive function in Case 2 was assumed to stem from the diffuse dysfunction of subcortical structures due to lesions.

It is widely recognized that anosmia and RBD are nonmotor symptoms that exist in the prodromal phase of PD, and almost all patients in our study had severe anosmia. RBD has been observed in some cases, and RBD and anosmia are relevant to the cognitive state of patients with PD [8, 28, 29]. Our patients' cognitive functions were noted to have decreased upon their study enrolment, and even our patients who were in their forties or fifties had poor cognitive functions at baseline. Decline in cognitive function has been reported to occur before the onset of schizophrenia, even during adolescence [30], or at early adulthood at 18 to 20 years old [31]. Furthermore, the patients' conditions worsen as they age [30]. Patients with schizophrenia appear to show the most significant decline at two key time points: the first prominent decline occurs prior to the first psychotic episode, and the second prominent decline begins at approximately 65 years of age [32]. The MMSE-detected deterioration in schizophrenia is less severe than that in Alzheimer's disease [32]. Of note, frontal lobe function was severely impaired in all patients in the current study. Dopamine therapy was important for not only parkinsonism but also cognitive function in Cases 1 and 3 during the follow-up period.

The limitations of this study are the small number of patients and the short study duration. We therefore cannot draw categorical conclusions about the observed cognitive characteristics. We also had limited information about the history of schizophrenia of the enrolled patients. Due to the long duration of their illness, we had to rely on self-reports and the ambiguity of the diagnostic criteria of schizophrenia limited our further analysis. As for our observations on Parkinson's disease, we cannot rule out potential genetic abnormalities that are presently unknown.

## 5. Conclusions

There is a possibility that PD patients are missed or misdiagnosed with schizophrenia with NIP and thus do not receive appropriate treatment. This study reports on a sample of schizophrenia patients with decreased uptake of dopamine on DAT scans who met the criteria of PD. Our patients benefitted from dopaminergic therapy without showing the deterioration of the positive symptoms of schizophrenia.

## Figures and Tables

**Table 1:** Clinical findings of the enrolled patients.

Case (sex)	1 (female)	2 (female)	3 (female)	4 (female)	5 (male)	6 (male)
*Age at schizophrenia diagnosis*						
	22	56	35	30	21	33

*D2 antagonist (mg)*						
Risperidone	4	—	4	—	—	9
Levomepromazine	5	—	5	—	—	9
Chlorpromazine	80	—		—	—	—

*Mood stabilizers (mg)*						
Valproic acid	—	500	600	—	—	1500

*Other antipsychotics (mg)*						
Quetiapine	—	125	—	—	50	600

*Age at parkinsonism onset*						
	37	68	68	65	46	76

*Time to diagnosis of PD from parkinsonism onset (years)*						
	5	4	4	10	1	2

*Age at enrolment*						
	44	74	74	75	53	80

*Parkinsonism dominant side*						
bilateral	Left	Bilateral	Bilateral	Left	Right	

*Follow-up (years)*						
	5	5	5	5	5	2

*Modified Hoehn and Yahr stage*						
At PD diagnosis	5	4	3	3	2	3
Baseline	2.5	3	3	3	2	2

*Levodopa (mg)*						
Baseline	300	200	300	600	150	0
End of study	500	400	400	600	300	0

*Dopamine agonist (mg)*						
Baseline	ROP-CR (12)	—	—	PRX-ER(1.13)	—	RTP (2.25)
End of study	ROP-CR (12)	—	—	PRX-ER(1.13)	—	RTP (2.25)

*Anticholinergic (mg)*						
Baseline	4	—	6	—	4	—
End of study	4	—	6	—	4	—

*Other anti-parkinsonian drugs (mg)*						
Baseline	—	ZNS (50)	Amantadine (100)	—	ZNS (50)	—
End of study	Entacapone (500)	ZNS (50)	Amantadine (100)	—	ZNS (50)	—

*MDS-UPDRS total*						
Baseline	70	48	26	82	33	91
End of study	60	69	61	110	47	

*MDS-UPDRS I*						
Baseline	12	2	8	22	3	31
End of study	8	15	14	14	5	38

*MDS-UPDRS II*						
Baseline	9	11	5	25	4	30
End of study	10	15	11	37	5	

*MDS-UPDRS III*						
Baseline	49	35	13	35	26	24
End of study	39	46	36	59	37	

*SBR of DAT scan (baseline)*						
Right	5.4	1.82	5.76	2.21	1.87	5.21
Left	6.19	1.83	6.04	2.73	1.86	4.43
AI %	13.7	0.3	4.7	21.1	0.6	16.2

*SBR of DAT scan (end of study)*						
Right	2.44	1.31	4.87	NA	NA	4.04
Left	2.94	0.7	5.37	NA	NA	4.23
AI %	18.6	60.5	9.8	NA	NA	4.5

*PDQ-39-Japan total*						
Baseline	55	16	82	92	29	76
End of study	40	62	40	79	NA	NA

*Moca-J*						
Baseline	26	19	21	10	19	NA
End of study	28	13	22	NA	NA	NA

*MMSE*						
Baseline	28	23	26	16	20	20
End of study	30	20	23	NA	NA	NA

*FAB*						
Baseline	15	12	9	7	8	NA
End of study	17	3	15	NA	NA	NA

*RBDQ-JP*						
Baseline	3	1	4	7	1	6
End of study	1	1	3	6	NA	NA

*WAIS verbal IQ*						
Baseline	84	114	94	NA	NA	NA
End of study	100	85	98	NA	NA	NA

*WAIS performance IQ*						
Baseline	69	65	87	NA	NA	NA
End of the study	101	53	90	NA	NA	NA

WAIS total IQ						
Baseline	74	91	93	NA	NA	NA
End of study	100	67	94	NA	NA	NA

*Neuropsychiatric Inventory severity*						
Baseline	7	4	14	NA	NA	NA
End of study	6	0	12	NA	NA	NA

*Neuropsychiatric Inventory distress*						
Baseline	4	2	10	NA	NA	NA
End of study	4	0	7	NA	NA	NA

*Noise pareidolia test (pareidolia)*						
Baseline	1	4	1	NA	NA	0
End of study	0	19	1	NA	NA	0

PD: Parkinson's disease, MDS-UPDRS: Movement Disorder Society-Unified Parkinson's Disease Rating Scale, SBR: specific binding ratio, DAT: dopamine transporter, PDQ-39-J: Parkinson's Disease Questionnaire-39 Japan, Moca-J: Montreal Cognitive Assessment-Japan, MMSE: Mini Mental Scale Examination, FAB: Frontal Assessment Battery, RBDQ-JP: Japanese version of the REM sleep behaviour disorder questionnaire, WAIS: Wechsler Adult Intelligence Scale. AI: asymmetry index, PRX-ER: pramipexole extended release, ROP-CR: ropinirole-controlled release, RTP: rotigotine transdermal patch, ZNS: zonisamide, and NA: not applicable.

**Table 2:** The NPI (Neuropsychiatric Inventory) subitems manifested in each patient and changes in these items from baseline to the end of the study.

Case	Subitem	Baseline			End of study		
		Frequency	Severity	total	Frequency	Severity	total
1	Agitation/Aggression	3	1	7/120	1	2	6/120
	Dysphoria/Depression	1	1		1	2	
	Irritability/Lability	3	1		1	2	

2	Apathy/Indifference	2	2	4/120	0	0	0/120

3	Delusions	1	2	14/120	0	0	12/120
	Hallucinations	2	1		0	0	
	Dysphoria/Depression	1	2		3	2	
	Apathy/Indifference	4	2		0	0	
	Anxiety	0	0		3	2

## Data Availability

The data used to support the findings of this study are available from the corresponding author upon request.

## References

[1] Fusar-Poli P., Meyer-Lindenberg A. (2013). Striatal presynaptic dopamine in schizophrenia, part II: meta-analysis of [(18)F/(11)C]-DOPA PET studies. *Schizophrenia Bulletin*.

[2] Madras B. K. (2013). History of the discovery of the antipsychotic dopamine D2 receptor: a basis for the dopamine hypothesis of schizophrenia. *Journal of the History of the Neurosciences*.

[3] Galoppin M., Berroir P., Soucy J. P. (2020). Chronic neuroleptic-induced parkinsonism examined with positron emission tomography. *Movement Disorders*.

[4] Tinazzi M., Cipriani A., Matinella A. (2012). [^123^I] FP-CIT single photon emission computed tomography findings in drug -induced parkinsonism. *Schizophrenia Research*.

[5] Postuma R. B., Berg D., Stern M. (2015). MDS clinical diagnostic criteria for Parkinson’s disease. *Movement Disorders*.

[6] Yokoi K., Nishio Y., Uchiyama M., Shimomura T., Iizuka O., Mori E. (2014). Hallucinators find meaning in noises: pareidolic illusions in dementia with Lewy bodies. *Neuropsychologia*.

[7] Sasai T., Matsuura M., Wing Y. K., Inoue Y. (2012). Validation of the Japanese version of the REM sleep behavior disorder questionnaire (RBDQ-JP). *Sleep Medicine*.

[8] Baba T., Kikuchi A., Hirayama K. (2012). Severe olfactory dysfunction is a prodromal symptom of dementia associated with Parkinson’s disease: a 3 year longitudinal study. *Brain*.

[9] Matsuda H., Murata M., Mukai Y. (2018). Japanese multicenter database of healthy controls for [^123^I]FP-CIT SPECT. *European Journal of Nuclear Medicine and Molecular Imaging*.

[10] Savica R., Grossardt B. R., Bower J. H., Ahlskog J. E., Mielke M. M., Rocca W. A. (2017). Incidence and time trends of drug-induced parkinsonism: a 30-year population-based study. *Movement Disorders*.

[11] Lieberman J. A., Stroup T. S., McEvoy J. P. (2005). Clinical antipsychotic trials of intervention effectiveness (CATIE) investigators. Effectiveness of antipsychotic drugs in patients with chronic schizophrenia. *New England Journal of Medicine*.

[12] Peluso M. J., Lewis S. W., Barnes T. R. E., Jones P. B. (2012). Extrapyramidal motor side-effects of first- and second-generation antipsychotic drugs. *British Journal of Psychiatry*.

[13] Parkinson Study Group (2002). Dopamine transporter brain imaging to assess the effects of pramipexole vs. levodopa on Parkinson disease progression. *JAMA*.

[14] Marek K., Innis R., van Dyck C. (2001). [123I] beta-CIT SPECT imaging assessment of the rate of Parkinson’s disease progression. *Neurology*.

[15] Pirker W., Djamshidian S., Asenbaum S. (2002). Progression of dopaminergic degeneration in Parkinson’s disease and atypical parkinsonism: a longitudinal beta-CIT SPECT study. *Movement Disorders*.

[16] Yamamoto H., Arimura S., Nakanishi A. (2017). Age-related effects and gender differences in Japanese health controls for [^123^I]FP-CIT SPECT. *Annals of Nuclear Medicine*.

[17] Roberts G., Lloyd J. J., Petrides G. S. (2019). 123 I-FP-CIT striatal binding ratios do not decrease significantly with age in older adults. *Annals of Nuclear Medicine*.

[18] Kordower J. H., Olanow C. W., Dodiya H. B. (2013). Disease duration and the integrity of the nigrostriatal system in Parkinson’s disease. *Brain*.

[19] Funayama M., Li Y., Tak-Hong T. (2008). Familial Parkinsonism with digenic parkin and PINK1 mutations. *Movement Disorders*.

[20] Takamura S., Ikeda A., Nishioka K. (2016). Schizophrenia as a prodromal symptom in a patient harboring SNCA duplication. *Parkinsonism & Related Disorders*.

[21] Nishioka K., Hashizume Y., Takanashi M. (2020). Pathological findings in a patient with alpha-synuclein p.A53T and familial Parkinson’s disease. *Parkinsonism & Related Disorders*.

[22] Kuusimäki T., Al-Abdulrasul H., Kurki S. (2021). Increased risk of Parkinson’s disease in patients with schizophrenia spectrum disorders. *Movement Disorders*.

[23] Smeland O. B., Shadrin A., Bahrami S. (2021). Genome-wide association analysis of Parkinson’s disease and schizophrenia reveals shared genetic architecture and identifies novel risk loci. *Biological Psychiatry*.

[24] Yoritaka A., Shimo Y., Hatano T., Hattori N. (2020). Motor/Non-motor symptoms and progression in patients with Parkinson’s disease: prevalence and risks in a longitudinal study. *Parkinsons Disease*.

[25] https://gskpro.com/content/dam/global/hcpportal/ja_JP/products-info/requip-cr/requip_cr-if.pdf.

[26] https://bij-kusuri.jp/products/attach/pdf/mra_lat0_375_if.pdf?msclkid=1820d0d2c79a11eca0f582ece0e9b4bd.

[27] https://image.packageinsert.jp/pdf.php?mode=1&yjcode=1169700S1025&msclkid=55c873d9c79c11ecbef25dfd996b4fe1.

[28] Gagnon J. F., Vendette M., Postuma R. B. (2009). Mild cognitive impairment in rapid eye movement sleep behavior disorder and Parkinson’s disease. *Annals of Neurology*.

[29] Roberts R. O., Christianson T. J. H., Kremers W. K. (2016). Association between olfactory dysfunction and amnestic mild cognitive impairment and alzheimer disease dementia. *JAMA Neurology*.

[30] Meier M. H., Caspi A., Reichenberg A. (2014). Neuropsychological decline in schizophrenia from the premorbid to the post onset period: evidence from a population-representative longitudinal study. *American Journal of Psychiatry*.

[31] Zammit S., Allebeck P., David A. S. (2004). A longitudinal study of premorbid IQ score and risk of developing schizophrenia, bipolar disorder, severe depression, and other nonaffective psychoses. *Archives of General Psychiatry*.

[32] Harvey P. D. (2014). What is the evidence for changes in cognition and functioning over the lifespan in patients with schizophrenia?. *Journal of Clinical Psychiatry*.

